# When connectivity depletes: the negative effects of enterprise social media use on employee well-being and work engagement

**DOI:** 10.3389/fpsyg.2026.1810219

**Published:** 2026-06-18

**Authors:** Da Eun Song, Seongcheol Kim

**Affiliations:** 1 Research Institute for Information and Culture, Korea University, Seoul, Republic of Korea; 2 College of Media and Communication, Korea University, Seoul, Republic of Korea

**Keywords:** boundary management, conservation of resources theory, enterprise social media, overload, well-being, work engagement

## Abstract

**Introduction:**

The rapid proliferation of digital technologies such as enterprise social media (ESM) has improved workplace collaboration by enabling seamless connectivity and real-time interaction across organizations. At the same time, it has introduced digital interruptions that demand constant responsiveness and digital presence from employees, which may be linked to adverse psychological and behavioral outcomes. Thus, a systematic examination of the negative effects of ESM on employee outcomes is needed.

**Methods:**

Grounded in the conservation of resources (COR) theory, this study used partial least squares structural equation modeling with a sample of 200 office workers in Korea to investigate how increased ESM usage is associated with multiple forms of overload and boundary management stress, and which specific forms were most directly related to employee outcomes such as well-being and work engagement. We also examined two variables—customized use of ESM features and perceived organization/team support—to test whether they moderate different forms of overload.

**Results:**

ESM usage was positively associated with higher levels of overload and boundary management stress. Interruption overload was significantly associated with lower work engagement, and boundary management stress was significantly associated with lower well-being. Customized use of ESM features and perceived organization/team support negatively moderated the relationship between ESM usage and boundary management stress.

**Conclusion:**

This study advances the technostress literature and offers practical implications for leveraging ESM to support sustainable digital work environments and mitigate adverse outcomes.

## 1 Introduction

The evolution of information technologies has revolutionized organizational communication, reshaping work systems and enabling seamless connectivity across traditional boundaries (Zhou and Mou, 2022). Previously, management information systems (MIS), used for data management and social functionalities like messaging, were limited in fostering dynamic interpersonal interactions. However, the advent of computer-mediated communication (CMC), with “masspersonal” platforms like email and instant messaging (High et al., 2023), evolved into enterprise social media (ESM), referring to any multifunctional, organization-bound digital platform that enables information and knowledge sharing, collaboration, and interpersonal interaction among employees (Leonardi et al., 2013; Leonardi, 2015). Features like visibility and persistence facilitate consistent content archiving, enhancing organizational communication (Nusrat et al., 2024; Y. Sun et al., 2022). In 2020, the onset of the COVID-19 pandemic further accelerated the adoption of digital work, and ESM became an increasingly essential tool to sustain productivity and foster connections among employees during physical separation (Ozimek, 2020).

Today, as digital platforms like ESM become more prevalent in the workplace, a growing body of research has explored their effects and implications. For example, prior studies have indicated that ESM improves various work-related outcomes for employees such as job performance (Ma et al., 2022), knowledge sharing (Leonardi, 2015), and creativity (Luqman et al., 2021; Zhang et al., 2024). However, these studies have primarily examined fragmented, individual-level outcomes, leaving a gap in our understanding of how ESM use relates to broader, more integrated organizational benefits, particularly by fostering work engagement. Meanwhile, recent scholarship has also drawn attention to the negative consequences of ESM, particularly the rise of digital interruptions and the associated stress in the workplace (Chen and Karahanna, 2018; Luqman et al., 2021; Safadi, 2024). Although some studies have examined both positive and negative consequences of ESM use, a more focused investigation is needed to identify which specific mechanisms of ESM-induced strain meaningfully translate into employee outcomes.

Addressing this gap, this study adopts a focused approach to investigate the negative effects of ESM usage on key employee outcomes such as well-being and work engagement. Work engagement in particular is a core factor in maintaining organizational productivity and competitiveness, which makes it a critical determinant of organizational outcomes (Bakker, 2017). With the widespread adoption of digital workplaces, organizations are now increasingly dependent on ESM for daily operations. Thus, understanding how ESM-related job demands affect employee efficiency and well-being has become a pressing concern. For example, while the time–space connectivity imposed by ESM facilitates convenient access to information and seamless communication, it can also be associated with overload and adverse psychological and behavioral outcomes for employees.

Grounded in the conservation of resources (COR) theory and job demands-resources model (JD-R), this paper investigates how ESM usage is associated with resource depletion among employees and, in turn, relates to their well-being and work engagement. We also explore two moderators that may be associated with attenuating the negative effects of ESM usage: (1) at the individual level, how employees manage work boundaries through the customized use of specific ESM features, and (2) at the organizational level, the role of perceived organization/team support. By examining these variables’ potential buffering effects, we provide practical implications for managing the risks associated with ESM use in digital workplaces and leveraging ESM’s potential for sustainable organizational outcomes.

Ultimately, this research furthers our understanding of how ESM usage relates to employee well-being and engagement in digital work settings. By differentiating among multiple forms of overload and boundary management stress, this study extends the technostress literature beyond the undifferentiated treatment of overload, suggesting that the dark side of ESM may operate through selective rather than general pathways of resource depletion. As digital work continues to dominate, such investigations are important for developing evidence-based strategies to ensure that employees thrive in digitally mediated workplaces.

The remainder of the paper is organized as follows. In Section 2, we discuss the transformative impact of communication within digital workplaces, focusing on the adoption of ESM and its influence on work dynamics. In particular, we examine how ubiquitous connectivity may be associated with resource depletion—conceptualized using overload variables and psychological responses—and investigate the related employee outcomes. Additionally, we identify two potential moderating factors in these relationships: the customized use of ESM features and perceived organization/team support. Based on this framework, we then develop research hypotheses. Section 3 details the research methodology, Section 4 presents our empirical findings, and Section 5 discusses results, conclusions, and implications.

## 2 Literature review

### 2.1 Communication in the digital workplace

#### 2.1.1 From CMC to ESM in the workplace

Advancements in digital technologies have led organizations to adopt a range of MIS to support workplace operations, including enterprise resource planning (ERP), customer relationship management (CRM), and communication platforms. Among these, communication-centric tools have gained prominence due to the critical role of communication in workplace tasks. Crucially, the adoption of specific communication technologies shapes the flow of information and stakeholder dynamics, thereby influencing productivity and engagement (Wu et al., 2023). CMC, a term originally introduced in the early 1970s to describe text-based computer messaging systems (High et al., 2023), now includes channels such as instant messaging (IM), email, and corporate blogs, which are features of modern organizational communication. CMC channels, characterized by “masspersonal” features that enable both interpersonal and mass communication (Sullivan and Carr, 2018), are vital in the workplace for promoting communication, relationships, and performance. However, research on CMC effectiveness has revealed mixed outcomes: while technologies like IM and email improve communication and work performance (Ou et al., 2013; Zhang et al., 2018), they are also associated with overload, burnout, and increased workplace stress (Barley et al., 2011; Stich et al., 2018).

Among CMC channels, enterprise social media (ESM) refers to platforms that are uniquely embedded in organizational contexts. Unlike traditional CMC technologies, ESM supports not only information sharing but also communication and collaboration among employees (Sun et al., 2022). Leonardi et al. (2013) explain, ESM enables employees to send messages, identify communication partners, post and edit content, and access others’ communications.

What distinguishes ESM from other CMC channels is its multifunctionality within a single platform. While communications via email or IM are typically confined to their participants, ESM offers visibility by enabling users to share their communication activities with others in the organization. In addition, the persistence of ESM ensures that the content remains accessible even after users log off. These features enable users to learn from others’ communication, reinforcing ESM’s role as a communication tool specialized for organizational use. As such, ESM has emerged as an effective channel for improving interaction, socialization, and collaboration within organizations, with an increasing number of firms adopting ESM to promote teamwork and knowledge integration. Following the COVID-19 pandemic, approximately 39.6% of employees reported using social media for work purposes (We Are Social and Meltwater, 2023). Popular ESM platforms include Slack, Microsoft Teams, and Yammer.

#### 2.1.2 The role of ESM in digital work environments

The COVID-19 pandemic fundamentally altered where and how work is conducted. Although the adoption of digital technologies was associated with the steady rise in remote work prior to the pandemic (Keightley et al., 2024), the need to mitigate virus transmission prompted many organizations to rapidly transition to remote work arrangements. As a result, this shift was linked to broader adoption of ESM as a vital means of enhancing communication and collaboration among geographically dispersed employees. Indeed, regular remote work has become the “new normal,” even after the pandemic. According to a report by McKinsey and Company (Alexander et al., 2021), today, many organizations are planning to adopt hybrid models that combine remote and in-office work, based on productivity gains observed during the pandemic. However, employees are also reporting heightened levels of anxiety and burnout. Thus, it is increasingly crucial to investigate the role of ESM in digital work environments.

With the widespread adoption of ESM, a substantial body of literature has emerged examining its outcomes, which remain mixed. On the positive side, numerous studies have demonstrated that ESM use may be associated with various benefits, for instance, improvements in job performance and creativity (Deng et al., 2021; Pitafi, 2025; Y. Sun et al., 2020), team and organizational effectiveness (Cao and Yu, 2019), and job satisfaction and individual productivity (Huang and Liu, 2017; Meske and Junglas, 2021).

Nevertheless, as these technologies expand employees’ capabilities and resources, they may also be associated with unintended negative consequences. Tarafdar et al. (2014) introduced the term “dark side” of IT to describe the potential risks of technology overuse, which may be linked to diminished individual, organizational, and societal well-being. The negative consequences of ESM use have been associated with technostress, perceived overloads, work interruptions, and emotional exhaustion (Marsh et al., 2022; Puranik et al., 2020; Stich et al., 2018; Sun et al., 2021).

As the adoption of ESM has accelerated, these platforms are now understood as more than just systems for knowledge transfer and task coordination: they also offer rich interactional and social affordances (Leonardi and Neeley, 2017). The decision to adopt ESM is no longer optional; it is fundamental for organizational operation. Thus, future research and practice must focus on the structural design and strategic use of ESM to optimize its effectiveness. Similarly, to remain competitive and achieve sustainable growth in the face of rapid market changes, organizations must prioritize employee work engagement and well-being (Bakker, 2017).

To this end, it is essential to examine the full spectrum of user experiences with ESM and its potential psychological consequences to address potential techno-pathologies in digital workplaces and support positive outcomes at both the individual and organizational levels. As ESM becomes an indispensable infrastructure in digitally mediated work environments, exploring not merely whether employees use it but *how* they use it and what affordances it provides is imperative for healthier and more productive digital work cultures (Safadi, 2024).

### 2.2 Conservation of resources theory and job demands-resources model

The proliferation of channels such as ESM has introduced new dynamics of connectivity in today’s communication environment. ESM is associated with a work culture characterized by ubiquitous connectivity and constant technical availability, which may be linked to employees internalizing a persistent sense of psychological engagement. Within this environment, users often feel compelled to remain cognitively connected to digital interactions even outside of traditional working hours (Johannes et al., 2021). Although the degree of this “connectedness mindset” may vary across individuals, those with a heightened sense of connectedness tend to be more vulnerable to overload and related psychological strain, especially in digital work settings (Li and Liu, 2025). Li and Liu (2025) argued that the increased use of ESM is linked to a continuous state of mental engagement, characterized by immediate responsiveness and continuous information monitoring. Therefore, while often perceived as a useful collaborative tool, ESM can also become a persistent psychological burden associated with the erosion of employees’ personal resources.

According to the COR theory, such erosion of personal resources is a fundamental trigger of stress, as individuals are inherently motivated to acquire, retain, and protect resources they perceive as valuable (Hobfoll, 1989; Luqman et al., 2021; Wang et al., 2020). In this context, resources include a broad array of entities, including tangible objects, personal characteristics such as self-esteem, energy, and other assets that are either intrinsically valuable or instrumental in obtaining or safeguarding other resources. COR theory explains stress in organized settings as arising from three conditions: perceived threat of resource loss, actual loss, or failure to gain resources after a loss (Hobfoll et al., 2018). It posits that universally valued resources make stress a sociocultural phenomenon; thus, it is relevant for analyzing workplace stress responses. Another important tenet of COR theory is the asymmetry between resource loss and gain, where individuals are more sensitive to the loss of resources than to equivalent gains. The fear of resource depletion can trigger defensive responses aimed at preserving remaining resources. In the organizational context, when excessive demands strain an individual’s coping capacity, this may be associated with resource depletion, reflected in outcomes such as burnout, emotional exhaustion, and psychological strain (Luqman et al., 2021; Wang et al., 2020).

This depletion of psychological and emotional resources aligns with the JD-R model, which classified all job characteristics into job demands and job resources, which jointly influence work-related outcomes. Job demands are the material, psychological, or organizational demands of work that require sustained effort-such as excessive workload and role conflict-and are typically associated with negative psychological and physiological responses (Mazzetti et al., 2023). In contrast, job resources refer to the psychological, social, or organizational resources that are inherently motivational in satisfying basic human needs and are available in the work environment, such as social support, job engagement, and reward. These resources may help reduce the costs associated with job demands and support employee growth and development (Demerouti et al., 2001; Hobfoll et al., 2003). From a JD-R perspective, the technologically enforced connectivity of ESM is linked to this depletion process by creating an environment where employees remain continuously accessible and responsive, which gradually cultivates a “connectedness mindset.” This persistent exposure to high job demands (e.g., excessive workload, emotional labor) is associated with the depletion of psychological and physical resources, ultimately related to exhaustion (Bakker and Demerouti, 2017).

Building on this perspective, this study investigates the consequences of resource depletion from ESM use. Specifically, we assess how ESM use is associated with individual-level cognitive and affective outcomes (e.g., well-being), as well as organizationally relevant outcomes (e.g., work engagement). By applying COR theory and JD-R model, this study provides a theoretical lens for understanding the cost of constant digital connectivity in modern workplaces and its implications for employee functioning and organizational effectiveness.

### 2.3 Perceived overload and boundary management stress

In this study, perceived overload in the digital workplace and boundary management stress arising from digital work environments are conceptualized as indicators of resource loss associated with ESM usage. We also examine these constructs as potential mediating variables linking employees’ ESM usage with outcomes such as well-being and work engagement.

There is a growing need to expand this understanding to include the multidirectional and persistent flows of communication and information across platforms. Lee et al. (2016) noted that the use of digital technologies may impose both behavioral and psychological burdens on individuals through mechanisms such as involuntary communication, the expansion of social networks, and information saturation. Particularly in the context of ESM, where interactions often transcend temporal and spatial boundaries, employees are especially vulnerable to various forms of overload.

Thus, we consider ESM use as associated with resource depletion and examine three forms of overload: information overload, communication overload, and interruption overload. In ESM contexts, information overload is defined as the perception that the volume of information transmitted exceeds an individual’s cognitive processing capacity (Chen and Wei, 2019). Communication or social overload refers to the perception of having to invest excessive social resources in others via ESM, often due to heightened social demands (Chen and Wei, 2019). Interruptions occur when stimuli or events disrupt the continuity of ongoing tasks, resulting in delays, fragmentation, or attention breakdown (Jett and George, 2003). Previous studies have identified overload as associated with role conflict (Carlson et al., 2017), work exhaustion (A. Chen and Karahanna, 2018), ESM-related exhaustion (Luqman et al., 2021), and stress (Marsh et al., 2024; Sun and Lee, 2022). Similarly, studies across various IS contexts have confirmed that overload is related to higher fatigue and lower psychological well-being and intentions to continue using the system (Sun et al., 2024; Yu et al., 2018).

In parallel, recent scholarship has sought to conceptualize psychological responses to digital work environments. For example, Keightley et al. (2024) identified two key responses to active ESM use in digital work environments: blurred work–life boundaries and intensified job demand. These have been associated with depletion of valuable psychological resources such as autonomy, flexibility, and time, and linked to negative cognitive, emotional, and behavioral outcomes (Marsh et al., 2022). According to boundary theory, boundaries that delineate specific domains-vary depending on the individual, who may perceive them as integrating or impermeable (Ashforth et al., 2000; Kreiner et al., 2009). However, since these boundaries are co-constructed accomplishments, discrepancies can occur between how individuals perceive their own boundaries and how others interpret them, potentially leading to conflict (Kreiner et al., 2009).

Active ESM engagement may blur the distinction between professional and personal spheres, which may increase the likelihood that an individual’s preferred boundaries will be violated or crossed by others. This can be explained through the concept of boundary violation, wherein excessive intrusion blurs the line between domains that should remain distinct, resulting in conflict (Hill et al., 2003). The constant temporal and spatial connectivity afforded by ESM is associated with heightened boundary management stress in digital work environments. Prior studies have also highlighted reduced job autonomy, increased work pressure, and a heightened sense of obligation as prominent characteristics of remote work settings, each representing essential personal resources needed to manage job demands (Keightley et al., 2024).

### 2.4 Outcomes of overload and boundary management stress in digital workplaces

Overload is a critical challenge associated with communication via ESM (Sun et al., 2024). Although ESM is related to improved access to information and promotes connectivity within organizational networks, it often requires employees to process excessive amounts of information and respond continuously to both work- and non-work-related demands, which may be related to adverse psychological and behavioral outcomes. This study investigates two of these outcomes, focusing on well-being and work engagement.

Overload and boundary management stress reflect a state of excessive energy depletion and may be associated with detrimental effects such as diminished well-being. In a general sense, well-being reflects individuals’ subjective assessments of their personal lives and is vital for organizational growth and sustainability (Spreitzer and Porath, 2012). Well-being is conceptualized through two perspectives: the hedonic view, which defines it as subjective happiness (subjective well-being), and the eudaimonic view, which sees it as the result of personal growth and self-realization (psychological well-being) (Zheng et al., 2015). In workplace contexts, Marsh et al. (2024) found that digital overload significantly associated with diminished employee well-being, emphasizing the need to address negative emotional experiences to foster organizational resilience. Boundary management stress has been linked to reduced emotional resilience, impaired stress recovery, and weakened capacity to maintain well-being (Keightley et al., 2024).

Work engagement, defined as the degree to which individuals invest themselves in their work roles (Kahn, 1990), is a key factor associated with organizational success. Previous studies have identified job resources and personal resources as key antecedents of work engagement. Despite the increasing reliance on ESM in digital workplaces, empirical research exploring its influence on work engagement remains limited. Among the studies that have examined this relationship, Sun et al. (2024) revealed that communication visibility through ESM was associated with overload and reduced employees’ work engagement. Their findings highlight the need for organizational policies that enable employees to manage or limit ESM functionalities to mitigate overload. Guo et al. (2026) similarly found that social-related ESM use was related to fatigue, which in turn negatively related to thriving at work, a construct closely related to work engagement. By incorporating work engagement as a key outcome variable, this study enhances understanding of ESM’s multifaceted role in shaping employee outcomes in digital work environments.

### 2.5 Perceived organization/team support

Several studies have demonstrated that organizational climate and the quality of supervisor–employee relationships can serve as moderators that alleviate the negative effects of technology use in the workplace (Marsh et al., 2022). Acquiring external resources can mitigate the negative effects associated with resource loss (Hobfoll et al., 2018). Thus, overload and the depletion of cognitive and emotional resources related to ESM use may be attenuated when alternative resources are made available. If individuals perceive strong organizational support, this may help to mitigate the negative consequences of ESM use.

For instance, Budnick et al. (2020) demonstrated that a family-supportive organizational climate can reduce the fear of missing out (FoMO) while at work and discourage employees from checking messages after work hours. Similarly, in the technostress literature, emotional support has been shown to reduce the perceived burden of techno-demands (Weinert et al., 2020). Khedhaouria et al. (2024) identified emotional social support—which includes support from family members, friends, colleagues, and even supervisors—as a key factor in reducing technostress and strain under remote work conditions. These results collectively suggest that a sense of community and positive interpersonal relationships, particularly with coworkers, can be essential in protecting against resource loss by excessive ICT use and assisting ESM users in digital work settings.

### 2.6 Hypothesis development

#### 2.6.1 Impact of ESM usage on perceived overload and boundary management stress

Based on prior IS research examining the relationship between individual usage behaviors and perceived overload (Chen and Karahanna, 2018; Sun et al., 2024; Tams et al., 2020; Yu et al., 2018), this study posits that employees’ excessive use of ESM during work hours may be associated with overload in digital work environments. Here, the concept of overload should not be limited to a single tool; rather, it must also be understood in the context of the broader flow of information and communication across multiple organizational platforms. In networked environments, where problem-solving and decision-making often require extensive information seeking via ESM, individuals may receive more information than they seek or need. Particularly in environments where individuals are connected not only with direct communicators but also with organization at large, considerable time and cognitive effort are required to extract relevant and useful information. Prior studies have identified the level of social media use as a key predictor of information overload (Yu et al., 2018). Accordingly, we propose the following hypothesis:

H1a: Employees’ ESM usage is positively associated with information overload.

Excessive ESM usage may also be associated with communication overload. Whereas information overload refers to the excessive volume of information that users attempt to process, communication overload is characterized by frequent, unplanned communication initiated by third parties. As the number of social connections via ESM increases, individuals may feel compelled to provide excessive communication and social support to those accessible through these online channels (Sun et al., 2024). This continuous stream of communication transmitted through multiple ties connected via ESM exposes individuals to persistent demands. In such environments, individuals may be required to exert additional and excessive efforts to meet the expected level of enacted support (Yu et al., 2018). Thus, we propose the following hypothesis:

H1b: Employees’ ESM usage is positively associated with communication overload.

Interruption overload refers to instances where work-related activities are interrupted excessively within a given period. Even brief, unanticipated interruptions can impose psychological burdens on employees (Luqman et al., 2021). Such interruptions may be associated with the depletion of time but also energy and mental vitality. In the context of ESM, the constantly connected work environment it creates allows users to be contacted regardless of what they are doing, placing them in situations where they must process continuous communication that is often unrelated to their current tasks. Such unsolicited communication, initiated by others rather than the individual, can disrupt concentration on work tasks and relate to frequent interruptions (Karr-Wisniewski and Lu, 2010). This is the basis of Hypothesis 1c:

H1c: Employees’ ESM usage is positively associated with interruption overload.

Meanwhile, active ESM engagement may often blur the distinction between professional and personal spheres, which may increase the likelihood that an individual’s preferred boundaries will be violated or crossed by others. This can be explained through the concept of boundary violation, wherein excessive intrusion blurs the line between domains that should remain distinct, resulting in conflict (Hill et al., 2003). The constant temporal and spatial connectivity afforded by ESM is associated with heightened boundary management stress in digital work environments. Previous studies have identified diminished job autonomy, increased work pressure, and heightened perceived obligation as salient features of remote work settings, each representing essential personal resources needed to manage job demands (Keightley et al., 2024). Accordingly, we propose the following hypothesis:

H1d: Employees’ ESM usage is positively associated with boundary management stress.

Despite these possible adverse effects, prior studies suggest that individuals may mitigate the negative consequences associated with ESM through personalizing their interaction with platform features and adjusting communication patterns to better fit their (Sun et al., 2021). According to Sun et al. (2021), individuals can actively employ strategies to regulate communication patterns in situations where constant connectivity makes personal boundaries increasingly ambiguous. When individuals consistently monitor and respond to communication demands in real time, they are more likely to experience heightened overload (Barley et al., 2011). Consequently, the strategic use of ESM, which refers to adjusting communication behavior to align with personal needs, can help manage communication processes more effectively. As with other information systems, ESM usage cannot be simply categorized into binary terms of “use” and “non-use”; rather, how individuals engage with the technology may be associated with the extent of resource depletion. Thus, we propose the following hypothesis:

H2: Employees’ customized use of ESM features moderates the relationship between ESM usage and (a) information overload, (b) communication overload, (c) interruption overload, and (d) boundary management stress, with increased levels of customization reducing these effects

#### 2.6.2 Outcomes of perceived overload and boundary management stress

Prior research on excessive technology use has identified overload as a key factor associated with negative outcomes (Yu et al., 2018). Overload is understood as arising from a misalignment between environmental demands and an individual’s coping abilities. Multiple studies have demonstrated that overload may be associated with lower well-being (Lutz et al., 2020) and reduced work engagement (Sun et al., 2024).

More specifically, diminished well-being has been linked to turnover intentions and absenteeism (Lee et al., 2023) and reduced organizational capabilities for innovation and sustained performance (De Neve et al., 2024), making the management of ESM-related overload a critical strategic priority. In a similar vein, Sun et al. (2024) demonstrated that communication overload has been associated with emotional exhaustion and reduced employee work engagement. Furthermore, boundary management stress has been associated with impaired ability to manage or recover from work stress, and linked to decreased well-being (Keightley et al., 2024).

The autonomy paradox describes how digital technologies offer remote work flexibility while being associated with expectations of constant availability, limiting employee autonomy (Kokshagina and Schneider, 2023). In flexible work settings, the ability to work across time and space requires individuals to balance competing work and personal demands, constantly reallocating resources like time and energy. Lutz et al. (2020) found that in setting with persistent technological accessibility, the pressure to stay available has been associated with reduced well-being during work and personal time. Based on these discussions, we propose the following hypotheses:

H3: Information overload is negatively associated with (a) well-being and (b) work engagement.

H4: Communication overload is negatively associated with (a) well-being and (b) work engagement.

H5: Interruption overload is negatively associated with (a) well-being and (b) work engagement.

H6: Boundary management stress is negatively associated with (a) well-being and (b) work engagement.

#### 2.6.3 Moderating effects of perceived organization/team support

Finally, this study examines whether perceived organization/team support may moderate the relationship between ESM usage and ESM-induced overload and boundary management stress. According to COR theory, individuals with greater resources are less sensitive to loss and more capable of acquiring additional resources (Hobfoll et al., 2018). Even if cognitive resources are depleted in association with overload, the availability of alternative resources such as positive interpersonal relationships within the organization can mitigate these effects. For example, prior studies have suggested that communication with leaders can provide employees with valuable resources and, in turn, improve their work behavior and performance (Davidson et al., 2017). Emotional social support (ESS) received from friends or colleagues has also been found to be effective in reducing technology-related overload and stress (Weinert et al., 2020). In the context of remote work specifically, research has shown that ESS moderates the relationship between technostressors and strain such that employees who perceive emotional support appraise stressors less negatively and experience reduced strain (Khedhaouria et al., 2024). Given these points, we propose our final hypothesis:

H7: Perceived organization/team support moderates the relationship between ESM use and (a) information overload, (b) communication overload, (c) interruption overload, and (d) boundary management stress, with higher levels of perceived organization/team support reducing these effects.

**Figure 1:**
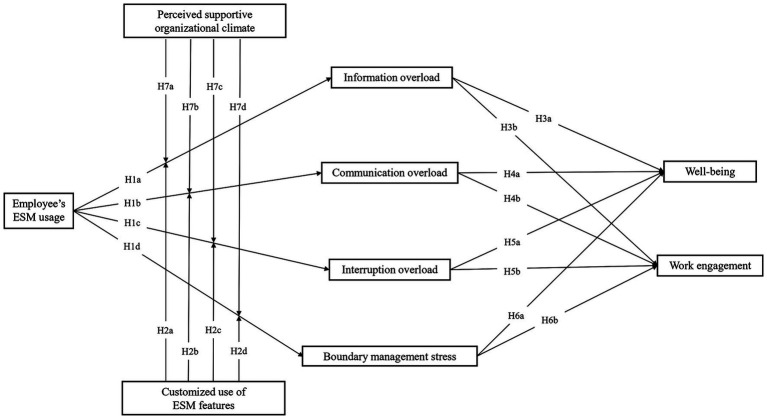
Research model.

The overall research model is described in Figure 1.

## 3 Methodology

### 3.1 Sample and data collection

An online survey was conducted from June 10 to June 13, 2025, in collaboration with Macromil Embrain, a professional survey agency in South Korea. Participants were limited to office workers who have experience with commonly used ESM in Korean corporate environments, such as Slack, Microsoft Teams, Naver Works, JANDI, Swit, and Flow. These platforms share key functionalities, such as real-time communication via topic- and team-based chat channels, file-sharing capabilities for various file types (e.g., images), and integration of multiple work-related services into a single platform. We also verified that the personally managed ESM features measured in our variables were available across all studied ESM platforms.

To ensure relevance to workplace settings, only individuals who reported working in remote or hybrid work arrangements and using these tools officially for work-related purposes within their organization were eligible to participate. We performed an *a priori* power analysis using G*Power (version 3.1.9.6) to determine the minimum required sample size to adequately test the hypotheses, and it indicated a minimum of 146 participants. The initial recruitment through Macromil Embrain yielded 150 valid responses, thereby exceeding this minimum requirement. However, because we had initially targeted a final sample size of 200 to improve analytic stability and account for potential response noise or invalid cases, an additional 50 participants were recruited via snowball sampling between June 10 and June 20, 2025. The final sample therefore consisted of 200 valid responses, including 150 from Macromil Embrain and 50 from snowball sampling.

To minimize social desirability bias, we asked participants to complete all survey items anonymously (Kim et al., 2023). Participants first completed items on work-related information, including the name of the ESM they primarily used, their average daily working hours, and their job position. Next, they responded to scales assessing perceived organization/team support, answered questions about ESM usage and their customized use of ESM features, and completed measures of overload, psychological responses, well-being, and work engagement. Finally, they provided demographic information, including gender and age.

The final sample comprised 56% male and 44% female participants, closely aligning with the national distribution of Korean office workers (52% male, 48% female) as reported by the Ministry of Employment and Labor (2024). The sample primarily included mid-level managers with an average age in their 30s to 40s, closely aligning with data on labor conditions by employment type in Korea. Although the demographic composition of our sample broadly aligns with national workforce statistics, the mixed recruitment strategy limits generalizability, and findings should be interpreted accordingly. To verify the homogeneity of the two subsamples, we conducted Chi-square tests and independent sample *t*-tests on key demographic variables and focal constructs; no significant differences were found between the groups (see Appendix 1). Table 1 summarizes the participants’ descriptive statistics.

**Table 1:** Participant demographics.

Variable	Frequency	Percent
Gender		
Male	113	56
Female	87	44
Age		
20–29	12	6
30–39	81	41
40–49	64	32
50–59	37	18
60+	6	3
ESM		
Slack	76	38
Teams	81	40
Works	27	14
Jandi	10	5
Flow	6	3
Job position		
Entry-level	18	9
Assistant manager	37	18
Manager	63	32
Senior manager	76	38
Executive-level	6	3
Average daily working hrs.		
≤6	3	1
7–8	108	54
9–10	68	34
11–12	16	8
>12	5	3
Department		
Planning, strategy	30	15
Marketing	29	15
Operations, CS	34	17
R&D, IT	52	26
Design	4	2
HR, general affairs	16	8
Finance, accounting	12	6
Other	23	11

ESM, enterprise social media.

### 3.2 Measurements

All constructs were modified from existing validated scales based on current research on ESM use and employee well-being and performance. All items were rated on a 7-point Likert scale ranging from 1 (“Strongly disagree” or “Do not use at all”) to 7 (“Strongly agree” or “Use very frequently”). The measurement items for ESM usage were adapted from Luqman et al. (2021); Zhong et al. (2012), and those for perceived organization/team support were adapted from Eisenberger et al. (2001). Customized use of ESM features was developed by the authors based on common functionalities shared across all studied ESM platforms. Prior to main data collection, a pilot test was conducted to verify that these features were actively used by office workers in practice, and items corresponding to unfamiliar or rarely used features were excluded. Unlike digital competence (general technology ability), self-regulation (internal behavioral control), or platform familiarity (passive exposure to a system), customized use of ESM features captures intentional, goal-directed behavioral adjustments made within the platform to manage communication demands and protect work-life boundaries. The items for information overload were adapted from Karr-Wisniewski and Lu (2010), communication overload from X. Chen and Wei (2019) and Karr-Wisniewski and Lu (2010), interruption overload from Chen and Karahanna (2018), boundary management stress from Keightley et al. (2024), well-being from Zheng et al. (2015), and work engagement from Schaufeli et al. (2006). Based on previous studies (Ayyagari et al., 2011; Chen and Karahanna, 2018; Marsh et al., 2024; Wang et al., 2020), this study also includes various control variables such as gender, age, average daily working hours, and job position. Gender and age are commonly included demographic variables, given that prior studies report these variables have been shown to impact technology usage behaviors, and they have been linked to inter-role conflict as well as related perceptions of a work environment (Chen and Karahanna, 2018; Ayyagari et al., 2011). Average daily working hours and job position were included because workload intensity and managerial responsibility may be associated with employees’ work engagement and well-being. All measurement items were translated into Korean by the authors and reviewed by the research team to ensure conceptual equivalence and linguistic appropriateness in the Korean workplace context. Table 2 presents a detailed list of the measurement items.

**Table 2:** Measurement items by construct.

Construct	Code	Measurement items	Source
ESM usage	EU1	I regularly use ESM to share content or information about work with coworkers.	Luqman et al. (2021); Zhong et al. (2012)
	EU2	I regularly use ESM to ask questions in my daily work.	
	EU3	I regularly use ESM to answer questions in my daily work.	
	EU4	I regularly use ESM to socialize in my daily work.	
Perceived organization/ team support	SC1	The organization/team takes pride in my accomplishments.	Eisenberger et al. (2001)
	SC2	The organization/team really cares about my well-being.	
	SC3	The organization/team strongly considers my goals and values.	
	SC4	The organization/team provides me with emotional support.	
Customized use of ESM features	CU1	When I take a break during working hours, I change my status to “away” or “on break.”	
	CU2	I have muted specific channels or messages.	
	CU3	I have used “focus mode” or “do not disturb” settings while working.	
Information overload	IO1	I am overwhelmed by the amount of information I have to process on a daily basis.	Karr-Wisniewski and Lu (2010)
	IO2	I am often distracted by the excessive amount of information available to me on ESM.	
	IO3	Usually, my problem is with too much information to synthesize instead of not having enough information.	
Communication overload	CO1	I am overwhelmed because ESM keeps me constantly connected to many people in real time.	X. Chen and Wei (2019); Karr-Wisniewski and Lu (2010)
	CO2	I waste my time responding to messages in collaboration environments that are work-related but not directly related to what I need to get done.	
	CO3	I am overwhelmed by social interactions with colleagues through ESM.	
Interruption overload	TO1	I am often overwhelmed by frequent notifications from ESM.	A. Chen and Karahanna (2018)
	TO2	I sometimes feel that I receive more messages on ESM than I can handle.	
	TO3	I sometimes find it difficult to concentrate on work due to receiving more notifications than I have time to deal with.	
Boundary management stress	BS1	I feel pressured to answer all work-related messages and requests right away.	Keightley et al. (2024)
	BS2	I find it difficult to completely disconnect from work after finishing my working day.	
	BS3	It is difficult for me to turn off the ESM when I finish the working day.	
	BS4	I sometimes feel guilty or anxious if I do not respond quickly to messages or requests received through ESM.	
	BS5	ESM makes me feel that work continues endlessly, blurring the boundaries between my work and personal life.	
	BS6	It is difficult for me to take breaks.	
Well-being	WB1	I feel satisfied with my life.	Zheng et al. (2015)
	WB2	I am in a good life situation.	
	WB3	Most of the time, I do feel real happiness.	
	WB4	I feel I have grown as a person.	
	WB5	I generally feel good about myself, and I am confident.	
	WB6	I have meaningful time with family and friends.	
Work engagement	WE1	At my work, I am bursting with energy.	Schaufeli et al. (2006)
	WE2	I can continue working for very long periods.	
	WE3	At my job, I am very mentally resilient.	
	WE4	I am enthusiastic about my job.	
	WE5	I find my work to be full of meaning and purpose.	
	WE6	I become immersed in my work.	
	WE7	I feel happy when I am working intensely.	

ESM, enterprise social media.

## 4 Data analysis and results

We used Smart PLS 3.0 to evaluate the measurement and structural models. For this study, we adopted partial least squares (PLS) as a soft modeling approach for structural equation modeling (SEM) to assess the appropriateness of the measurement constructs and examine the hypothesized associations among latent variables (Hair and Alamer, 2022; Kim et al., 2023). PLS-SEM’s methodological advantages include robustness to small sample sizes and flexibility regarding data distribution, as it does not require the assumption of normality and is more suitable for exploratory studies (Richter et al., 2022; Wong, 2013). Thus, we considered PLS-SEM to be suitable for this study’s analytic framework.

### 4.1 Measurement model

We assessed the items’ internal consistency reliability using Cronbach’s alpha and composite reliability (CR), and these values indicate good internal reliability. The Cronbach’s alpha values of all constructs exceed 0.70 (0.724–0.941) (Wong, 2013), and the CR of each construct exceeds 0.7 (0.844–0.947) (Table 3).

**Table 3:** Reliability and validity results.

Construct/item	Factor loading	AVE	CR	Cronbach’s *α*
ESM usage		0.851	0.958	0.941
EU1	0.922			
EU2	0.934			
EU3	0.944			
EU4	0.889			
Perceived organization/team support		0.693	0.900	0.863
SC1	0.861			
SC2	0.778			
SC3	0.860			
SC4	0.828			
Customized use of ESM features		0.644	0.844	0.724
CU1	0.747			
CU2	0.793			
CU3	0.863			
Information overload		0.808	0.926	0.881
IO1	0.920			
IO2	0.938			
IO3	0.918			
Communication overload		0.768	0.909	0.849
CO1	0.869			
CO2	0.902			
CO3	0.858			
Interruption overload		0.856	0.947	0.916
TO1	0.920			
TO2	0.938			
TO3	0.918			
Boundary management stress		0.672	0.925	0.902
BS1	0.765			
BS2	0.867			
BS3	0.834			
BS4	0.784			
BS5	0.862			
BS6	0.800			
Well-being		0.712	0.937	0.918
WB1	0.874			
WB2	0.866			
WB3	0.889			
WB4	0.747			
WB5	0.870			
WB6	0.807			
Work engagement		0.700	0.942	0.929
WE1	0.809			
WE2	0.894			
WE3	0.806			
WE4	0.894			
WE5	0.856			
WE6	0.750			
WE7	0.836			

AVE, average variance extracted; CR, composite reliability; ESM, enterprise social media.

We evaluated convergent validity using the average variance extracted (AVE) and factor loadings. A commonly accepted threshold for AVE is 0.5 or higher, while a factor loading of 0.7 or higher is typically considered adequate. As indicated in Table 3, the AVE of all constructs exceeds 0.50 (0.644–0.856), and the factor loadings of all construct items exceed 0.70, indicating sufficient convergent validity.

We evaluated discriminant validity using heterotrait–monotrait (HTMT) ratio of correlation. All HTMT values are less than 0.9 (Henseler et al., 2015), which further suggests good discriminant validity (Table 4). Because all the variables were collected from the same source, we assessed potential common method bias using the full collinearity assessment approach proposed by Kock (2015). All inner VIF values were below the threshold of 3.3, indicating that common method bias is unlikely to threaten the validity of this study. Additionally, Harman’s single factor test was conducted by entering all measurement items into an exploratory factor analysis and constraining the solution to one factor. The single factor accounted for 34.89% of the total variance, which is well below the 50% threshold, suggesting that common method bias is less likely to pose a serious threat to the validity of this study.

**Table 4:** HTMT ratios.

Construct	EU	SC	CU	IO	CO	TO	BS	WB	WE
EU									
SC	0.053								
CU	0.463	0.279							
IO	0.765	0.092	0.449						
CO	0.694	0.137	0.328	0.808					
TO	0.804	0.263	0.306	0.752	0.819				
BS	0.686	0.157	0.296	0.673	0.591	0.762			
WB	0.230	0.556	0.072	0.267	0.347	0.425	0.377		
WE	0.205	0.516	0.094	0.283	0.281	0.434	0.345	0.807	

EU, enterprise social media use; SC, perceived organization/team support; CU, customized use of ESM features; IO, information overload; CO, communication overload; TO, interruption overload; BS, boundary management stress; WB, well-being; WE, work engagement.

### 4.2 Structural model

**Figure 2:**
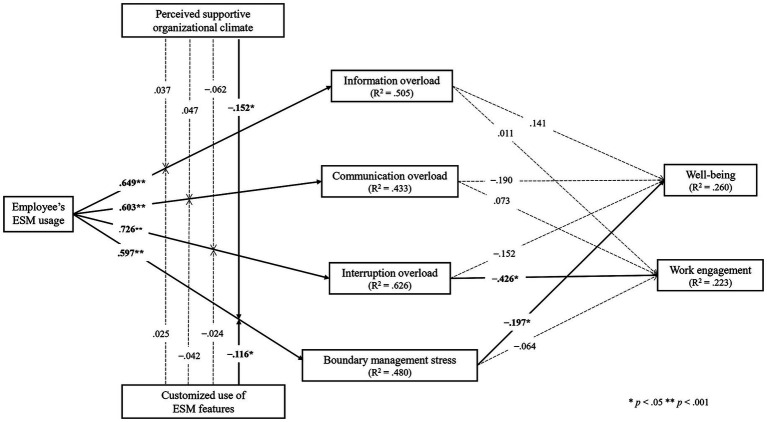
Structural model testing results.

We used the bootstrapping approach with 200 cases and 5,000 resamples to test the structural model, and the results are presented in Table 5 and Figure 2.

**Table 5:** Hypothesis testing results.

Hypothesis	Path	*β*	*t*	Result
H1a	EU → IO	0.649^**^	13.325	Supported
H1b	EU → CO	0.603^**^	10.226	Supported
H1c	EU → TO	0.726^**^	16.845	Supported
H1d	EU → BS	0.597^**^	11.627	Supported
H2a	EU × CU → IO	0.025	0.432	Rejected
H2b	EU × CU → CO	−0.042	0.668	Rejected
H2c	EU × CU → TO	−0.024	0.426	Rejected
H2d	EU × CU → BS	−0.116^*^	2.012	Supported
H3a	IO → WB	0.141	1.328	Rejected
H3b	IO → WE	0.011	0.108	Rejected
H4a	CO → WB	−0.190	1.712	Rejected
H4b	CO → WE	0.073	0.622	Rejected
H5a	TO → WB	−0.152	1.286	Rejected
H5b	TO → WE	−0.426^*^	3.467	Supported
H6a	BS → WB	−0.197^*^	2.105	Supported
H6b	BS → WE	−0.064	0.605	Rejected
H7a	EU × SC → IO	0.037	0.711	Rejected
H7b	EU × SC → CO	0.047	0.843	Rejected
H7c	EU × SC → TO	−0.062	1.394	Rejected
H7d	EU × SC → BS	−0.152^*^	2.972	Supported

EU, enterprise social media use; SC, perceived organization/team support; CU, customized use of ESM features; IO, information overload; CO, communication overload; TO, interruption overload; BS, boundary management stress; WB, well-being; WE, work engagement.

*p < 0.05, **p < 0.001.

Among the control variables, job position was significantly and positively associated with well-being and work engagement. Average daily working hours were significantly and negatively associated with communication overload and well-being. However, gender and age have no significant effects on boundary management stress, well-being, work engagement, or any of the overload variables.

We evaluated the *R*-square statistics for the dependent variables to assess how well the model explains and predicts the data (Figure 2). These values reflect the strength of relationships and the substantive effects of the associated latent variables. The *R*-square values for boundary management stress, well-being, and work engagement were 48, 26, and 22.3%, respectively. Regarding the overload variables, the *R*-square values for information overload, communication overload, and interruption overload were 50.5, 43.3, and 62.6%, respectively.

Next, the results indicate significant positive associations between ESM use and information overload (*β =* 0.649*, p* < 0.001), communication overload (*β =* 0.603*, p* < 0.001), interruption overload (*β =* 0.726*, p* < 0.001), and boundary management stress (*β =* 0.597*, p* < 0.001), thereby supporting H1. H3 through H6 examined the negative effects of overload variables and boundary management stress on well-being, and work engagement. Among these, two hypotheses (H5b, H6a) were supported. Specifically, interruption overload was significantly and negatively associated with work engagement (*β = −*0.426*, p* = 0.002), while boundary management stress was significantly and negatively associated with employee well-being (*β = −*0.197*, p* = 0.046). The remaining hypotheses were not supported.

**Figure 3:**
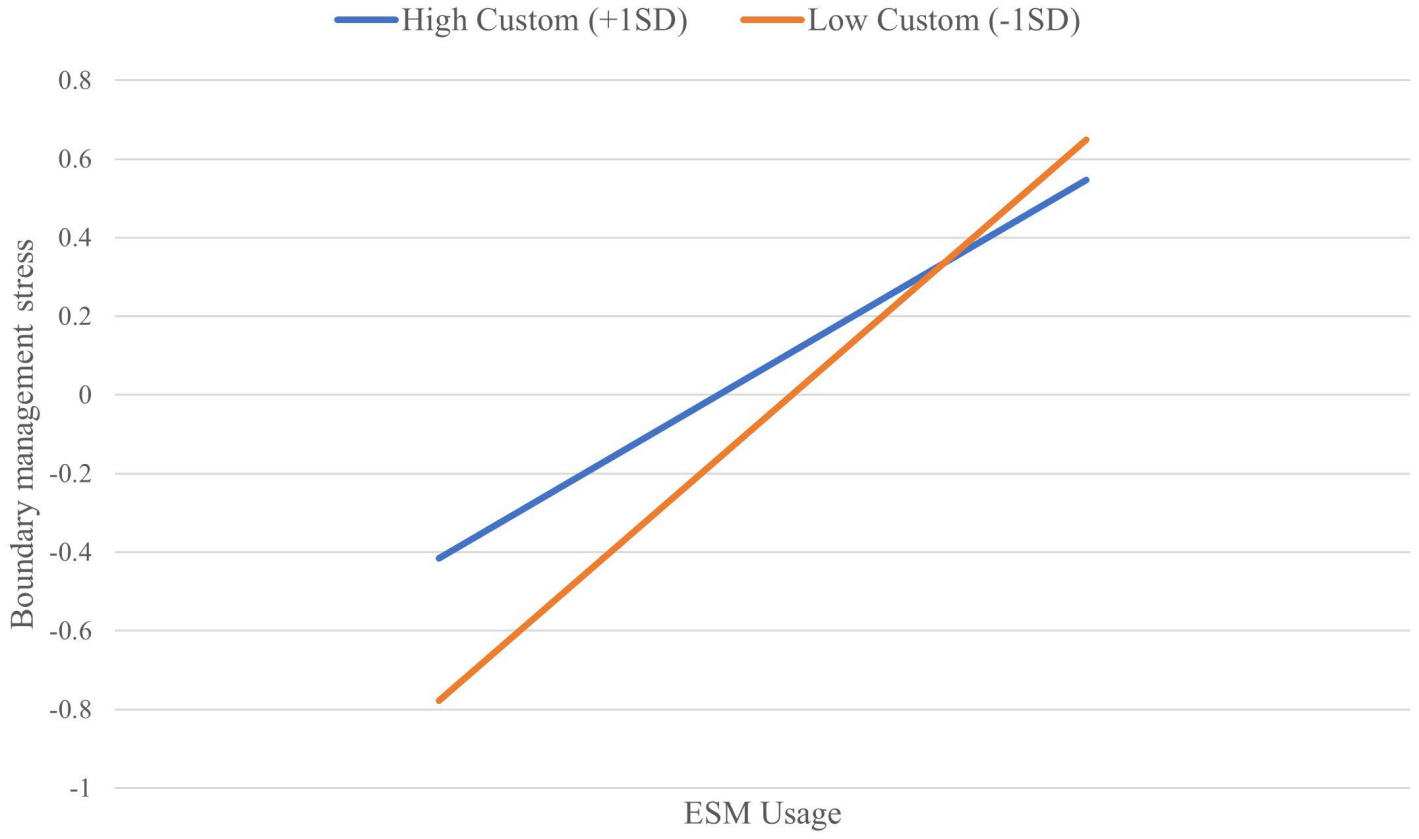
Moderating effect of customized use of ESM features on the relationship between ESM usage and boundary management stress.

**Figure 4:**
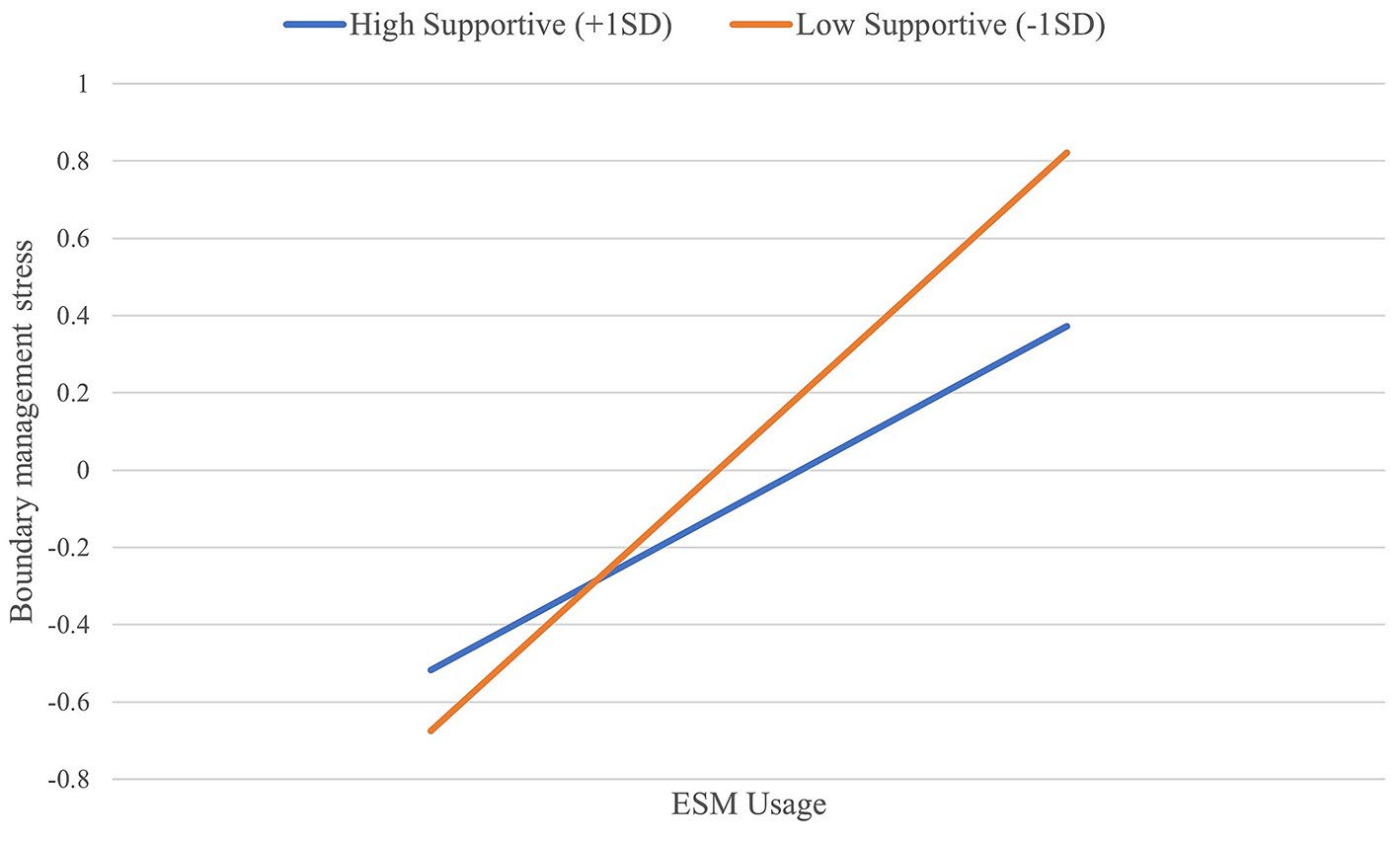
Moderating effect of perceived organization/team support on the relationship between ESM usage and boundary management stress.

We also hypothesized that the customized use of ESM features and perceived organization/team support moderate the relationships between ESM use, the overload variables, and boundary management stress. Of the eight hypotheses examining moderating effects, only H2d and H7d were supported. Specifically, customized use of ESM features negatively moderated the relationship between ESM use and boundary management stress (*β =* −0.116*, p* = 0.044). Similarly, perceived organization/team support negatively moderated the relationship between ESM use and boundary management stress (*β =* −0.152*, p* = 0.002). To further illustrate the supported moderation effects, interaction plots were constructed using ±1 SD of the moderator variables. As shown in Figure 3, the positive relationship between ESM usage and boundary management stress was weaker when customized use of ESM features was high (1 + SD) than when it was low (−1SD). As shown in Figures 4, a similar pattern was observed for perceived organization/team support, such that the relationship between ESM usage and boundary management stress was weaker when perceived organization/team support was high (+1SD) than when it was low (−1SD).

Table 6 presents the specific indirect effects of ESM usage on well-being and work engagement through each mediator, with 95% bias-corrected bootstrap confidence intervals. Only two indirect effects were statistically significant: ESM usage was negatively associated with work engagement through interruption overload (*β = −*0.310, 95% CI [−0.491, −0.130]), and negatively associated with well-being through boundary management stress (*β = −*0.117, 95% CI [−0.233, −0.009]).

**Table 6:** Specific indirect effects of ESM usage on employee outcomes.

Path	*β*	95% CI	Result
EU → IO → WB	0.091	[−0.033, 0.245]	Rejected
EU → IO → WE	0.007	[−0.124, 0.139]	Rejected
EU → CO → WB	−0.115	[−0.243, 0.022]	Rejected
EU → CO → WE	0.044	[−0.087, 0.203]	Rejected
EU → TO → WB	−0.110	[−0.281, 0.055]	Rejected
EU → TO → WE	−0.310^*^	[−0.491, −0.130]	Supported
EU → BS → WB	−0.117^*^	[−0.233, −0.009]	Supported
EU → BS → WE	−0.038	[−0.163, 0.087]	Rejected

EU, enterprise social media use; SC, perceived organization/team support; CU, customized use of ESM features; IO, information overload; CO, communication overload; TO, interruption overload; BS, boundary management stress; WB, well-being; WE, work engagement.

*p < 0.05, **p < 0.001.

Structural VIFs for all paths were below the threshold of 3.3, indicating no multicollinearity concerns. Effect sizes (*f*^2^) indicated that ESM usage showed large effects on all four strain variables (*f*^2^ = 0.538–1.059), while the effects of strain variables on outcomes were generally small (*f*^2^ = 0.002–0.072), with interruption overload showing the largest effect on work engagement (*f*^2^ = 0.072). As a robustness check, recruitment source (panel vs. snowball) was included as a control variable linked to all endogenous constructs, and the structural model was re-estimated. As shown in Appendix 2, all key relationships remained consistent in significance and direction, with only negligible changes. These results, together with the subsample comparison in Appendix 1, suggest the mixed recruitment strategy did not introduce meaningful bias.

## 5 Discussion and conclusion

### 5.1 Summary and interpretation

This study develops a framework to examine how different forms of overload and boundary management stress are associated with employees’ psychological and behavioral outcomes, including well-being, and work engagement, in the context of ESM usage. Additionally, we assess the moderating role of the customized use of ESM features and perceived organization/team support on the relationships between (1) ESM usage and different forms of overload and (2) ESM usage and boundary management stress. Our formulated relationships have been developed based on COR theory.

Initially, we investigated the mechanism of resource depletion associated with ESM use in the workplace. Our findings reveal that employees’ extensive use of ESM was significantly associated with information overload, communication overload, interruption overload, and boundary management stress. These results align with prior research highlighting the adverse consequences of ESM use (Chen and Karahanna, 2018; Luqman et al., 2021). In other words, incessant demands from colleagues, excessive information, and the necessity of simultaneous communication with multiple coworkers through ESM may be associated with impaired ability to concentrate on core tasks and depletion of personal resources. This aligns with the central tenet of COR theory that the diversion of resources such as time and energy to competing domains diminishes the resources available to individuals (Hobfoll et al., 2018).

Then, we examined how this resource depletion relates to employees’ psychological and behavioral outcomes. Contrary to expectations, information overload showed no significant association with well-being, or work engagement. Similarly, communication overload showed no effect on psychological or behavioral outcomes. These findings diverge from prior studies suggesting that information and communication overload could precipitate diminished well-being and work engagement (Eliyana et al., 2020; Marsh et al., 2024; Sun et al., 2024). In other words, in this study, the resource depletion associated with information and communication overload in the context of excessive ESM use did not directly translate into broader psychological or behavioral consequences.

Given that information overload was not significantly associated with psychological or behavioral outcomes, prior studies on workplace fear of missing out (FoMO) may offer some insight. Marsh et al. (2024) found that information-related FoMO was associated with employees’ mental health, yet was not significantly related to information overload itself. This raises the possibility that FoMO may shape how employees perceive and respond to information demands, rather than directly translating into negative outcomes. However, as FoMO was not measured in this study, this interpretation warrants direct empirical examination.

Furthermore, the Korean work context, characterized by hierarchical structures and high work intensity, may partly account for this pattern, as employees may be more accustomed to managing high information demands. In contrast, employees in more individualistic workplace cultures, where autonomy and work–life balance are emphasized, may respond differently to similar levels of ESM-induced overload. However, as cultural factors were not directly assessed in this study, this explanation should be treated as tentative and requires cross-cultural empirical validation. Moreover, communication overload was not significantly associated with the outcomes studied. Yu et al. (2018) suggested that socially oriented requests are less directly tied to core work tasks and may therefore carry a relatively lower psychological burden. While this reasoning is consistent with our findings, the underlying mechanism was not captured in our measures, and further research is needed to substantiate this interpretation.

By contrast, interruption overload and boundary management stress were found to be significantly associated with reduced work engagement and well-being, respectively. In this study, interruption overload is defined as breaks in the continuity of an enduring task associated with “techno-invasion” (Luqman et al., 2021), while boundary management stress is conceptualized as reactions to the erosion of work–life boundaries linked to such techno-invasion. Boundary management stress most acutely reflects the erosion of work–life boundaries associated with the ubiquitous nature of ESM. While ESM is related to greater autonomy in terms of time and location, it also comes with expectations of constant responsiveness across multiple communication channels (Barley et al., 2011). Such expectations may be related to reduced psychological detachment from work and impaired emotional recovery and well-being (Büchler et al., 2020). Guo et al. (2026) similarly found that employees with higher levels of psychological detachment showed a weaker negative association between fatigue and thriving at work, suggesting that the ability to mentally disengage from work-related demands may serve as a protective resource against ESM-related well-being costs. In this regard, the flexibility that ESM provides frequently becomes an unspoken requirement to always be reachable, which may be related to heightened interruption overload and blurred work–life boundaries essential for well-being.

Similarly, continuous demands from colleagues via ESM may be linked to reduced work engagement. Interruptions have long been recognized as being associated with reduced productivity, higher error rates and longer task completion times. When interruptions exceed manageable thresholds, they may substantially undermine work engagement and performance (Chen and Karahanna, 2018). In the digital workplace, incessant connectivity and the expectation of perpetual availability have been linked to an expanded scope of work and heightened interruption overload. Excessive interruptions have been associated with hindered timely response to critical tasks, which may relate to missed responsibilities and reduced employees’ pride and enthusiasm for their work. The negative outcomes of interruption overload are primarily reflected in diminished work engagement, which may be related to resource depletion within these domains. By contrast, boundary management stress is predominantly associated with well-being, likely due to resource depletion specific to these areas. The differential pattern of these negative outcomes can be explained by the varying scope of resource depletion across these domains.

This study also explores coping strategies for technostress by examining two moderating variables. Our findings indicate that both variables negatively moderate the relationship between ESM use and boundary management stress. Safadi (2024) noted that the outcomes of ESM use are heavily contingent on its design and users’ attitudes, with asymmetric engagement among users enabling cognitive benefits. Thus, coping strategies hinge not only on whether ESM is used but also on how it is utilized. This study provides evidence that customized use approaches may serve as a potential mechanism for addressing the erosion of work–life boundaries by demonstrating that adaptive usage patterns may be associated with reduced negative consequences of ESM. Similar patterns have been observed with job autonomy, which reflects individual’s ability to regulate how and when they perform their work tasks, and has been associated with reduced stress responses in digitally intensive work environments (Guo et al., 2026; Wei et al., 2025).

In addition, perceived organization/team support provides an organizational-level answer to resource depletion. While organizational support strategies have been extensively examined in the IS literature, the moderating effects of emotional social support on technostress and strain have produced mixed findings (Khedhaouria et al., 2024). Nevertheless, this study finds that perceived organization/team support was related to a significant moderating effect on the relationship between ESM use and boundary management stress. These findings indicate that a supportive environment, characterized by collegial intimacy and team cohesion, may buffer the adverse psychological reactions associated with excessive ESM use. Consequently, cultivating such a climate may represent a potential organizational-level strategy for addressing the adverse effects of technostress in the digital workplace.

However, neither variable moderated the relationship between ESM use and the examined forms of overload. These results may reflect the structural nature of overload, which is often associated with the inherent characteristics of digital communication platforms and the volume of work demands rather than by individual coping or organizational support alone. In other words, overload may be less sensitive to discretionary usage behaviors or ambient organizational climate and more strongly tied to systemic or task-related factors that lie beyond users’ immediate control. Prior studies have suggested that technological coping strategies may help reduce the adverse association between technostress and performance and moderate the relationship between overload and strain (Galluch et al., 2015; Pirkkalainen et al., 2019). Similarly, high-quality leader–employee relationships have been shown to buffer the relationship between overload and work–family conflict (Harris et al., 2013). However, research on the boundary conditions associated with the attenuation of technostress outcomes remains underdeveloped, particularly regarding the effectiveness of specific strategies in managing overload. We hope that this study can serve as a starting point for future research to explore strategies for mitigating ESM-related technostress.

### 5.2 Implications

Our findings highlight several theoretical and practical implications. From a theoretical perspective, this study advances the theoretical discourse on technostress by differentiating among three distinct types of overloads in the context of ESM: information overload, communication overload, and interruption overload. Importantly, not all forms of overload were equally associated with negative outcomes: only interruption overload was significantly associated with reduced work engagement. This differential pattern suggests that overload may not function as a monolithic construct, and points to the value of distinguishing among its forms in future research. Then, we explore their differential associations with psychological and behavioral outcomes. By employing COR theory, this study further highlights how boundary management stress may be associated with diminished well-being. Taken together, these findings suggest that the negative effects of ESM operate through selective rather than general pathways, and future theorizing should reflect these pathway-specific patterns rather than assume all forms of overload are similarly associated with negative outcomes.

Finally, this study elucidates the role of customized use of ESM features and perceived organization/team support as potential buffering factors, specifically in relation to boundary management stress. However, these moderating effects were limited to the ESM usage - boundary management stress pathway and were not observed for the overload variables, suggesting that individual-and organizational-level strategies may have a more bounded scope than initially hypothesized. These findings provide preliminary evidence that targeted interventions may help address the association between technostress and employee well-being.

Our findings also offer several practical implications. First, for corporate management, the adverse associations of excessive ESM use, particularly interruption overload, highlight the need for balanced usage policies. Although ESM can be a critical platform for organizational operations, sustaining organizational growth may benefit from prioritizing employee well-being and maintaining their engagement with both the organization and their work (Sun et al., 2024). While many organizations emphasize prompt responsiveness and discourage communication delays, they may also consider establishing operational norms that enable technology to support, rather than hinder collaboration. Such efforts may contribute to fostering a healthy work environment that supports productivity and employees’ well-being.

Second, for ESM platform providers and developers, the finding suggests the value of designing functionalities that enable users to achieve psychological detachment during both work and non-work hours, which may help address the detrimental associations of constant connectivity with mental health. Through targeted interventions, employees should be empowered to focus on tasks or disengage from work as needed. Additionally, employees may be encouraged to understand and utilize ESM functionalities to maintain focus on work tasks while preserving boundaries outside of work.

Third, team managers should understand the essential role of fostering perceived organization/team support. Managers should cultivate a culture of emotional well-being and mutual support to ensure that employees feel emotionally connected and confident in the support provided by their colleagues and the organization as a whole.

Lastly, our findings may offer tentative directions for future research on regulatory approaches to technostress. For example, future work could explore whether approaches such as digital rest periods or supportive frameworks for psychologically sustainable work systems may help address the adverse associations of ESM use.

### 5.3 Limitations and future research

Regarding limitations, first, the cross-sectional design limits the study’s ability to establish temporal precedence or rule out reciprocal relationship. Although the JD–R model guides the framework, the design does not allow for causal inference. For instance, employees who experience higher exhaustion may report greater overload and boundary stress, reversing the assumed direction. Future research should employ longitudinal or experimental methods, such as multi-wave panels or experience-sampling methods, to track changes in ESM use, job demands, and outcomes over time and provide stronger evidence on directionality.

Second, our sample was limited to employees in South Korea, which may limit generalizability to other cultural or national contexts. South Korea’s unique work environment, characterized by comparatively long working hours, a hierarchical organizational culture, and advanced digital infrastructure, likely influences ESM use and its outcomes. Similarly, cultural dynamics, such as high-context communication norms and collectivism, may shape ESM-related experiences. To better understand how cultural factors affect overload and the dark-side outcomes, future research should adopt a cross-cultural perspective and compare ESM use and its effects across diverse cultural and organizational contexts. Additionally, although participants were screened to include those in remote or hybrid work arrangement, the survey did not assess remote work intensity or after-hours ESM use. These contextual factors were therefore not directly examined and should be addressed in future research.

Third, participants were recruited through two rounds of data collection from diverse organizations, which may have introduced variability in the employees’ ESM experiences. Differences in organizational structures, ESM adoption levels, and workplace norms likely influenced how employees interacted with ESM, which could have affected the consistency of the observed outcomes. Furthermore, all data were collected via self-reported surveys from a single source at a single point in time, which raises concerns about common method bias. Although full collinearity assessments were conducted, these statistical checks do not fully resolve this limitation. Future research should examine employees within a single organizational context or compare across distinct organizational cultures to better isolate contextual effects on ESM-related outcomes.

Fourth, this study does not differentiate between types of technologies, such as mobile- versus desktop-based ESM platforms. Given that technology-specific characteristics—–including the immediacy of feedback, notification patterns, and portability—may influence the intensity and perception of overload, future studies should categorize ESM usage by platform type to investigate how platform-specific attributes affect overload and boundary management stress.

Fifth, while all measurement items were translated into Korean by the authors and reviewed by the research team to ensure conceptual equivalence, formal back-translation procedures were not conducted, which may limit the cross-linguistic validity of the adapted scales. Future research should employ rigorous translation protocols, including back-translation and expert review, to strengthen measurement equivalence across languages.

Sixth, although a pilot test was conducted to verify feature familiarity during the development of the customized use of ESM features scale, the pilot test procedure and item-retention criteria were not formally documented. This represents a measurement limitation, and future research should employ more rigorous scale development procedures with formal pilot testing and item-retention criteria.

## Data Availability

The raw data supporting the conclusions of this article will be made available by the authors, without undue reservation.
