# Supplementary material for: When connectivity depletes: the negative effects of enterprise social media use on employee well-being and work engagement
Source: Front Psychol. 2026 Jun 18;17:1810219. doi: 10.3389/fpsyg.2026.1810219 (PMC13323711; doi:10.3389/fpsyg.2026.1810219)
Supplement: Supplementary file 1 [file Supplementary_file_1.docx]

Appendix 1. Comparison of panel and snowball subsamples

| Variable | Panel (N = 150) | Snowball (N = 50) | Statistic | *p* |
| --- | --- | --- | --- | --- |
| Demographics |  |  |  |  |
| Gender (Male%) | 54.7% | 62.0% | χ² *=* 0.82 | .365 |
| Age | – | – | χ² *=* 4.47 | .346 |
| Job position | – | – | χ² *=* 2.02 | .731 |
| Focal Constructs | Mean (SD) | Mean (SD) | *t* |  |
| EU | 4.62 (1.45) | 4.44 (1.43) | 0.78 | .438 |
| SC | 4.88 (1.07) | 4.78 (1.20) | 0.36 | .721 |
| CU | 3.63 (1.33) | 3.55 (1.11) | 0.57 | .569 |
| IO | 4.46 (1.46) | 4.36 (1.41) | 0.42 | .679 |
| CO | 3.93 (1.42) | 4.12 (1.49) | −0.80 | .422 |
| TO | 4.07 (1.43) | 4.29 (1.64) | −0.94 | .350 |
| BS | 3.40 (1.05) | 3.43 (1.07) | −0.19 | .851 |
| WB | 4.69 (1.10) | 4.44 (1.17) | 1.34 | .183 |
| WE | 4.41 (1.12) | 4.31 (1.29) | 0.53 | .594 |

*Note.* EU = enterprise social media use, SC = perceived organization/team support, CU = customized use of ESM features, IO = information overload, CO = communication overload, TO = interruption overload, BS = boundary management stress, WB = well-being, WE = work engagement.

Appendix 2. Comparison of structural model estimates

| Path | Original Model β (*t*) | With Recruitment Control β (*t*) |
| --- | --- | --- |
| EU → IO | 0.649^**^ (13.325) | 0.647^**^ (13.227) |
| EU → CO | 0.603^**^ (10.226) | 0.593^**^ (9.991) |
| EU → TO | 0.726^**^ (16.845) | 0.720^**^ (16.450) |
| EU → BS | 0.597^**^ (11.627) | 0.599^**^ (11.674) |
| EU × CU → BS | −0.116^*^ (2.012) | −0.121^*^ (2.057) |
| TO → WE | −0.426^*^ (3.467) | −0.394^*^ (3.078) |
| BS → WB | −0.197^*^ (2.105) | −0.202^*^ (2.141) |
| EU × SC → BS | −0.152^*^ (2.972) | −0.152^*^ (2.968) |

*Note.* EU = enterprise social media use, SC = perceived organization/team support, CU = customized use of ESM features, IO = information overload, CO = communication overload, TO = interruption overload, BS = boundary management stress, WB = well-being, WE = work engagement. Recruitment source (panel = 1, snowball = 2) was included as a control variable.

* *p* < .05, ** *p* < .001.
